# Properties and Possibilities of Using Biochar Composites Made on the Basis of Biomass and Waste Residues Ferryferrohydrosol Sorbent

**DOI:** 10.3390/ma17112646

**Published:** 2024-05-30

**Authors:** Katarzyna Wystalska, Mariusz Kowalczyk, Tomasz Kamizela, Małgorzata Worwąg, Magdalena Zabochnicka

**Affiliations:** Faculty of Infrastructure and Environment, Czestochowa University of Technology, Brzeźnicka 60A, 42-200 Częstochowa, Poland; mariusz.kowalczyk@pcz.pl (M.K.); tomasz.kamizela@pcz.pl (T.K.); malgorzata.worwag@pcz.pl (M.W.); magdalena.zabochnicka@pcz.pl (M.Z.)

**Keywords:** waste iron sorbent, pyrolysis, Fe-biochar composites, adsorption, methylene blue, amoxicillin, diclofenac, heavy metals, plant growth

## Abstract

Biochar enriched with metals has an increased potential for sorption of organic and inorganic pollutants. The aim of the research was to identify the possibility of using biochar composites produced on the basis of waste plant biomass and waste FFH (ferryferrohydrosol) containing iron atoms, after CO_2_ capture. The composites were produced in a one-stage or two-stage pyrolysis process. Their selected properties were determined as follows: pH, ash content, C, H, N, O, specific surface area, microstructure and the presence of surface functional groups. The produced biochar and composites had different properties resulting from the production method and the additive used. The results of experiments on the removal of methylene blue (MB) from solutions allowed us to rank the adsorbents used according to the maximum dye removal value achieved as follows: BC1 (94.99%), B (84.61%), BC2 (84.09%), BC3 (83.23%) and BC4 (83.23%). In terms of maximum amoxicillin removal efficiency, the ranking is as follows: BC1 (55.49%), BC3 (23.51%), BC2 (18.13%), B (13.50%) and BC4 (5.98%). The maximum efficiency of diclofenac removal was demonstrated by adsorbents BC1 (98.71), BC3 (87.08%), BC4 (74.20%), B (36.70%) and BC2 (30.40%). The most effective removal of metals Zn, Pb and Cd from the solution was demonstrated by BC1 and BC3 composites. The final concentration of the tested metals after sorption using these composites was less than 1% of the initial concentration. The highest increase in biomass on prepared substrates was recorded for the BC5 composite. It was higher by 90% and 54% (for doses of 30 g and 15 g, respectively) in relation to the biomass growth in the soil without additives. The BC1 composite can be used in pollutant sorption processes. However, BC5 has great potential as a soil additive in crop yield and plant growth.

## 1. Introduction

The United Nations Climate Change Conference in Paris stated the ultimate need to stop the increase in CO_2_ concentration in the atmosphere due to its catastrophic impact on the climate. According to the Kyoto Protocol, six anthropogenic greenhouse gases (GHGs), such as carbon dioxide, methane, nitrous oxide, hydrofluorocarbons, perfluorocarbons and sulfur hexafluoride, influence global warming [1]. Almost 80% of the volume of all greenhouse gases emissions in the EU in 2021 was represented by carbon dioxide [2]. Motorization, burning fossil fuels and many industries such as, for example, steel, aluminum, cement and petroleum industries emit mainly carbon dioxide [3]. Reducing carbon dioxide emissions has recently become a priority action in counteracting climate change. Effective solutions that will reduce the emission of this gas into the atmosphere are still being sought. The lowering of carbon dioxide concentrations in the atmosphere could be realized by several methods such as, for example, utilization of clean energy sources and capture of carbon dioxide gas and its storage or use for other purposes. CO_2_ could be captured during absorption, cryogenic distillation, membrane-based separation, adsorption, etc. Many inorganic or organic sorbents could be used for carbon capture and post-process wastes could be used as materials for other technologies [4]. The Lithuanian company INECO has patented an FFH sorbent designed to remove contaminants from wastewater [5]. As part of the project “Capture of Carbon Dioxide by Innovative Sorbent InnoCO2Sorbent” (No. EUREKA/InnoCO2Sorbent/2/2021), research is being carried out on the use of this sorbent to capture CO_2_ from exhaust gases. FFH (ferryferrohydrosol) is a compound produced in the process of hydrolysis and polymerization of iron ions in aqueous electrolyte solutions that contains iron atoms in various ionic states, composed mainly of iron hydroxide (Fe(II)), ferric hydroxide and ferric oxide (Fe(III)) [5]. It is characterized by high reactivity and adsorption properties [5]. The absorption of carbon dioxide with FFH into a stable and environmentally friendly compound is a promising method. After the CO_2_ capture process by FFH, waste material will remain. Therefore, research is underway to determine potential methods of managing these waste FFH (referred to as FFHCO2 in the rest of the article).

The expansion of modern production technologies, increasing the potential of car transport, has contributed to the release of many inorganic pollutants (including heavy metals) into the environment [6]. In addition, increased consumption of pharmaceuticals [7] causes pollutants present in wastewater that contaminate water and soils, which is a global problem that poses a direct threat to the environment [8,9,10,11].

Various methods are used to remove contaminants from aqueous solutions [11]. However, adsorption is considered one of the most effective techniques for removing contaminants, due in part to its simplicity, and ability to remove different types of contaminants [6]. However, the production of some adsorbents may involve high financial outlays [12,13]. Therefore, there is still a need for cheap adsorbents [7,14].

Waste can be used to produce adsorbents [15]. Transforming waste into products with added value is consistent with the assumptions of the circular economy. Due to its specific properties, biochar can be used in many different environmental processes, related, among others, to the removal of pollutants from water, soil or gases [14,16,17,18,19]. As indicated by [20], such features of biochar, including large specific surface area, porous structure, a large number of surface functional groups (including carboxyl, hydroxyl and phenolic), mineral components and hydrophobicity [16], determine its use as an adsorbent for removing contaminants from aqueous solutions. This activity is beneficial both in ecological and economic terms.

Biochar is produced in the pyrolysis process, at elevated temperatures, with limited access to oxygen. The use of slow pyrolysis allows for a higher yield of the solid phase (biochar) compared to other products [21,22], and the process itself is treated as more environmentally friendly and perceived as a sustainable technology [16]. The quality of the obtained biochar is influenced by the type of raw material processed [23], process temperature, heating time and reaction time [22,24,25,26]. High pyrolysis temperature increases the specific surface area of biochars, microporosity and hydrophobicity [27]. As the pyrolysis temperature increases, the importance of pore-filling mechanisms and hydrophobic interactions [26] in terms of sorption mechanisms also increases. According to researchers [26], the optimal temperature range used for the production of biochars is 500–800 °C, but the higher the pyrolysis temperature, the higher the energy consumption of the process.

Waste plant biomass [28], manure or sewage sludge are often used as a raw material for the production of biochar [29,30]. Biochars of lignocellulosic origin usually have a larger specific surface than biochars made from sewage sludge or chicken manure. The presence of large amounts of ash in biochars of fecal origin clogs the pores with mineral substances and blocks the development of the specific surface [21,31]. Therefore, in our research, waste plant biomass, i.e., sawdust remaining from wood processing, was used to produce biochar.

Research results from many authors indicate that not only the use in adsorption processes but also the addition of biochar enriches the soil [32], increases its fertility [33] and the bioavailability of nutrients [34,35], and increases pH [36]. It is also a very good solution in the process of carbon sequestration in the soil [37]. The authors [33] described that biochar can increase the water-holding capacity and availability of water in the soil. The use of biochar may therefore have a positive impact on the growth and yield of plants [38,39,40,41]. The specific properties of biochars also allow their use for the recultivation of degraded areas [42]. However, research on the enrichment of biochar is still being conducted, because, as indicated by [43], it has been proven that enriched biochars with improved physicochemical properties improve the biological, physical and chemical properties of the soil, as well as the growth and yield of plants compared to unenriched biochar.

Typically, the original properties of biochars are also not sufficient to remove contaminants from aqueous solutions at a satisfactory level. To increase the effectiveness of sorption processes, biochars are often modified [7,12,44,45] using water vapor, acids, alkalis [46,47], magnetic modification [48], impregnation with various substances, including materials with the presence of iron [49,50] or by creating composites/nanocomposites [51].

According to [26], enriching biochar with metallic materials affects its physical and chemical properties. Research results presented in the literature indicate that the addition of metallic substances can improve the efficiency of the use of biochars for the removal of various types of organic and inorganic substances [26,48,52,53]. Magnetic biochar composites have the potential to remove contaminants such as antibiotics, organic dyes, pesticides, phenols and organochlorine compounds from solutions [54]. The use of Fe-biochar composites for methylene blue dye removal was described by [55], their research results showed that introducing an iron oxide particle onto the biochar surface improved the adsorption capacity of methylene blue. However [56] investigated the behavior of iron-functionalized biochar for microplastic removal. Viglasowa et al., 2020 [57] described reduced nitrogen species from an aqueous solution using biochar modified with Fe and Mg. Wang et al., 2020 [58] used biochar enriched with iron particles for ammonium adsorption. The authors [59,60] showed in their studies that the Fe-biochar composite has the potential to remove selenium from aqueous solutions. Similar trends were described by [61,62], who showed the ability of iron-modified biochar to adsorb arsenic at the level of 98%. Many researchers have described the use of modified biochars [7], including those modified with iron [63,64]. Mortazavian et al., 2019 [46] indicated that Fe-biochar composites are a promising adsorbent for removing heavy metals from water, and examined the effectiveness of this composite as Fenton’s reagent. Composites based on tea stems and iron solution showed the potential to degrade organic pollutants via the Fenton reaction [65]. Qiu et al., 2022a [16] reported that Fe_2_O_3_-impregnated biochar prepared from waste tea stems and FeSO_4_ combined with calcination showed improved phenol, ciprofloxacin, norfloxacin and enrofloxacin decomposition performance in the short term. The addition of Fe(III) ions significantly increased the efficiency of bisphenol A and phenanthrene removal and increased the stability of biochar [66].

The methods for producing biocarbon-based composites include initial processing of biomass with additional raw materials and then pyrolysis, or subjecting the biomass to pyrolysis and then processing with additional raw materials and/or secondary pyrolysis [14,47,51,52,61,62,67] produced Fe-biochar composites by impregnating biochar samples with iron compounds and post-processing them. The authors [68] produced an iron oxide-biochar composite using a ferric chloride solution and pomelo peel in a one-step method. Similarly, in their research, refs. [55,69] used straw impregnated with iron compounds and then subjected to the pyrolysis process.

According to [16] metals such as iron, aluminum, magnesium and manganese are most often used to create biocarbon composites. Typically, the production process of biochar composites uses commercial substances, i.e., compounds of various metals. In our research, waste FFHCO2 containing iron was used, and to compare the efficiency of pollutant sorption, iron salts were also used to produce composites.

The production of biochar-based composites has the advantage that the product combines the properties of biochar and additional material [70]. As indicated by [71], these products demonstrate high efficiency in removing pollutants, but the potential risks related to their stability and toxicity require further research.

In summary, the main goal of our research was to evaluate the feasibility of using biochar-Fe composites to remove organic and inorganic contaminants from aqueous solutions and use them as a soil improver. Waste plant biomass and waste FFHCO2 were used to produce the composites. Due to the lack of information in the literature on the use of composites produced from this type of waste, composites impregnated with commercial iron compounds were produced for comparison of properties and performance. Selected properties of biocarbon and produced composites were determined. Recognition and evaluation of the use of composites as adsorbents and soil additives in the process of plant growth and yield were carried out. This recognition will help direct the conduct of further detailed research.

## 2. Materials and Methods

### 2.1. Materials and Reagents Used in the Research

#### 2.1.1. Substrates for the Production of Biochars and Fe-Biochar Composites

The basis for the production of composites was waste plant biomass, i.e., sawdust remaining after processing pine wood, taken from a sawmill in southern Poland. The sawdust was dried and sifted through a 5 mm sieve.

**Figure 1:**
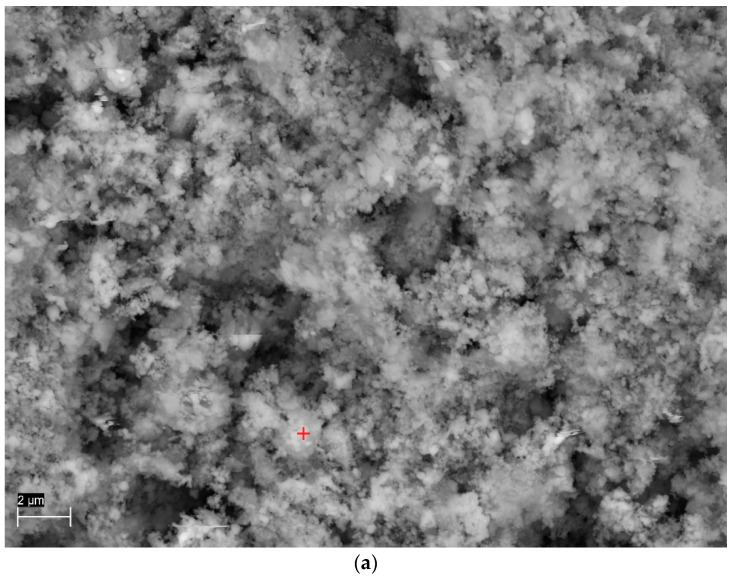

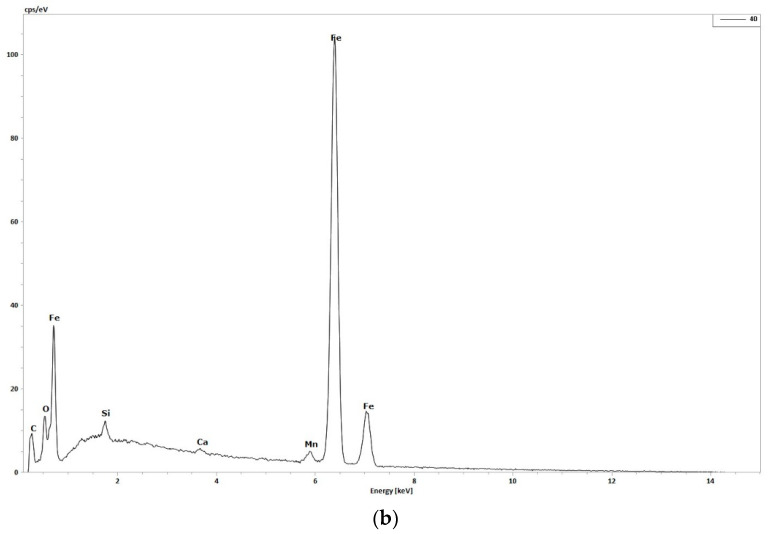
Results of electron microscopy analysis of FFHCO2: (**a**) microstructure with the EDS analysis site marked, (**b**) results of point EDS analysis.

FFHCO2, waste from the CO_2_ capture process by FFH, was used as an additional material for the production of composites. The collected FFHCO2 had a pH of 7.9 and a dry matter content of 11.22%. The spot analysis of the chemical composition (Figure 1) showed the presence of an intense peak indicating the presence of iron in FFHCO2.

#### 2.1.2. Reagents and Materials

Iron salts were used to produce the following Fe-biochar composites: iron (II) chloride 4·H_2_O (pure for analyses), purchased from Chemipur (Piekary Śląskie, Poland), and iron (III) chloride 6·H_2_O (pure for analyses), purchased from Eurochem BGD Sp. z o. o. (Tarnów, Poland). Pharmaceuticals used for sorption experiments, i.e., diclofenac sodium and amoxicillin trihydrate, were purchased from Glentham Life Sciences. Substances containing heavy metals, including ZnCl_2_, CdCl_2_ ·2.5 H_2_O and Pb(NO_3_)_2_, were purchased from Chemipur (Piekary Śląskie, Poland). Methylene blue was purchased from Hadron Scientific.

The soil for testing was collected from fields in southern Poland, where weak, podsolic soils dominate. New Zealand spinach seeds were purchased from PlantiCo—Hodowla i Nasiennictwo Ogrodnicze Zielonki Sp. z. o. o.

### 2.2. Research Methodology

#### 2.2.1. Parameters of the Pyrolysis Process

The production of biochar (marked with the symbol B later in the article) and the thermal treatment of materials for composites were carried out in the process of slow pyrolysis under established process parameters. The process temperature was 600 °C, the heating time was 1.5 h and the reaction time was 1 h. The process was carried out in a two-half furnace, type PRW S100x780/11 (manufacturer—Czylok Company from Jastrzębie-Zdrój, Poland) in an atmosphere of inert gas (nitrogen) with a flow rate of 5 L·m^−1^.

#### 2.2.2. Manufacturing of Composites

Two types of composites were used in the research: waste composites based on sawdust and FFHCO2 and composites based on sawdust and FeCl_2_ + FeCl_3_ solution. The composites were produced using two methods. The first method involved pyrolysis of biochar impregnated with iron compounds. In the second method, raw biomass (sawdust) was impregnated with iron compounds and then transformed in the pyrolysis process. Composites with the addition of commercial iron compounds were produced in order to compare the effects of operation with waste composites.

Composites with the addition of FFHCO2 were manufactured according to the following procedure: (i) Sawdust was impregnated with FFHCO2, and 80 g of sawdust + 400 g of waste was mixed and left for 24 h, and then dried at 105 °C. The dried material was pyrolyzed under established process conditions. After cooling down and reaching ambient temperature, the composites were rinsed with boiling distilled water (1 L of water/100 g of material) and then dried in a dryer at 105 °C. This composite was designated BC1. (ii) Biochar was impregnated with FFHCO2: 40 g of biochar and 400 g of waste were mixed and left for 24 h, and then dried in an oven at 105 °C. The dried material was pyrolyzed under established process conditions. After cooling down and reaching ambient temperature, the composites were rinsed with boiling distilled water (1 L of water/100 g of material) and then dried in a dryer at 105 °C. This composite was designated BC2.

Composites with the addition of a FeCl_2_ + FeCl_3_ solution were produced analogously, replacing FFHCO2 with a FeCl_2_ + FeCl_3_ solution. The iron salt solution was prepared by dissolving 5.523 g of FeCl_2_·4 H_2_O and 15.02 g of FeCl_3_·6 H_2_O in 400 mL of distilled water. (iii) An amount of 80 g of sawdust and 400 mL of the solution were mixed and left for 24 h. then dried at 105 °C. The dried material was pyrolyzed under established process parameters. After cooling down and reaching ambient temperature, the composites were rinsed with boiling distilled water (1 L of water/100 g of material) and then dried in a dryer at 105 °C. This composite was designated BC3. (iv) Biochar was impregnated with a FeCl_2_ + FeCl_3_ solution: 40 g of biochar and 400 mL of the solution were mixed and left for 24 h and then dried at 105 °C. The dried material was pyrolyzed under established process parameters. After cooling down and reaching ambient temperature, the composites were rinsed with boiling distilled water (1 L of water/100 g of material) and then dried in a dryer at 105 °C. This composite was designated BC4.

For the purposes of the plant growth experiment, another type of composite was produced—biochar impregnated with FFHCO2 and left in an incubator at 28 °C until dry. The mass fractions of the individual components were the same as described above. This composite was designated BC5.

#### 2.2.3. Methodology of Physico-Chemical Analyses

The dry matter content in FFHCO2 was determined in three replicates in accordance with PN-EN 12880:2004 Characterization of Sewage Sludge—Determination of Dry Residue and Water Content. pH was determined using the potentiometric method (triple measurement) using a pH meter. pH value measurement was made by placing 5 g of the sample in three, individual beakers and then adding distilled water to each of them (50 mL). The beakers were shaken for 10 min and then infiltrated. The ash content in biochar and composites was determined in accordance with PN-EN ISO 18122:2016-01 Polish version, Solid Biofuels—Determination of Ash Content. Total carbon content (TC) in the BC5 composite was determined by Multi N/C, Analytykjena—the high-temperature incineration with detection IR. PN-ISO10694:2002—Soil Quality—Determination of Organic Carbon Content and Total Carbon Content After Dry Combustion (Elemental Analysis). The concentration of heavy metals in aqueous solutions was determined in accordance with the PN-EN ISO 11885:2009 standard Water Quality—Determination of Selected Elements by Optical Emission Spectrometry with Inductively Excited Plasma (ICP-OES). The study of the morphology of the FFHCO2 surface and the chemical composition in micro-areas was performed using an SEM SIGMA VP scanning electron microscope from ZEISS (Jena, Germany), which is connected to an EDS UltraDry energy-dispersive X-ray spectrometer. The tested sample was placed on a graphite disc. In order to study the grain morphology, the sample was additionally sprayed with gold. Micro-area photos of biochar and composite samples were taken using a scanning electron microscope, a Hitachi SU3500 (Tokyo, Japan). Elemental analysis (determination of nitrogen, carbon, hydrogen and oxygen content) was performed using the dynamic combustion method in the FLASH 2000 analyzer (three combustions), (Waltham, MA, USA). The BET (Brunner–Emmett–Teller theory) surface area was indicated by the ASAP 2420 (Haan, Germany). The ASAP 2420 analyzer measures single and multi-point specific surface areas and the size and distribution of pore solid samples. Surface functional groups were identified by Attenuated Total Reflectance Fourier transform infrared spectroscopy (FTIR).

#### 2.2.4. Methodology of Sorption Experiments

##### Methylene Blue (MB) Sorption

Weighted portions of 0.1 g, 0.20 g, 0.3 g and 0.4 g were prepared from the prepared composites and biochar, which were then mixed with 20 mL of prepared methylene blue (MB) solutions with initial concentrations (*C*_1_): 50 mgL^−1^, 75 mgL^−1^ and 100 mgL^−1^. The mixtures were prepared in three repetitions. The prepared samples were shaken on a laboratory shaker for 24 h, then left in static conditions for another 24 h. The final concentration of MB (*C*_2_) in the solutions was measured using a UV-VIS spectrophotometer (DR 500 HACH Lange, Loveland, CO, USA) at a wavelength of 664 nm.

The percentage of MB removal from the solution was calculated according to the following formula:

(1) $$W=\frac{C_{1}-C_{2}}{C_{1}}\times 100\%$$

where

*C*_1_—initial concentration of MB solution, mgL^−1^;
*C*_2_—MB solution concentration during equilibrium, mgL^−1^.

##### Sorption of Pharmaceuticals: Amoxicillin and Diclofenac

Weighted portions of 0.05 g, 0.1 g, 0.2 g and 0.4 g were prepared from the prepared composites and biochars. Solutions were prepared from pharmaceuticals with the following concentrations: 10 mg·L^−1^, 50 mg·L^−1^ and 100 mg·L^−1^. The prepared weighted portions were poured with 25 mL of a solution with the appropriate concentration of the pharmaceutical. The bottles with the mixture were shaken on a laboratory shaker (150 rpm) for 24 h. Then, it was left in static conditions for 6 h. The final concentration (*C*_2_) of diclofenac in the solutions was measured using a UV-VIS spectrophotometer (DR 500 HACH Lange, Loveland, CO, USA) at a wavelength of 276 nm, and the final concentration of amoxicillin at a wavelength of 228 nm.

The percentage of pharmaceutical removal from the solution was calculated according to the following formula:

(2) $$W=\frac{C_{1}-C_{2}}{C_{1}}\cdot 100\%$$

where

*C*_1_—initial concentration of the pharmaceutical drug solution, mgL^−1^;
*C*_2_—concentration of the pharmaceutical drug solution during equilibrium, mgL^−1^.

##### Sorption of Heavy Metals: Zn, Cd and Pb

Weighted portions of 0.8 g were prepared from the prepared composites and biochar. Solutions with a metal ion concentration of 50 mg·L^−1^ were prepared. Biochar samples were placed in glass bottles and filled with the prepared solution of the tested metal ions. The bottles with the suspension were shaken on a laboratory shaker (150 rpm) for 24 h. The mixtures were then filtered through a hard filter (Waterman, Columbus, OH, USA) and a syringe filter (45 μm). The content of the elements Zn, Cd and Pb was determined in accordance with PN-EN ISO 11885:2009 Water Quality—Determination of Selected Elements by Optical Emission Spectrometry with Inductively Excited Plasma (ICP-OES).

#### 2.2.5. Methodology of the Pot Experiment

The soil intended for the experiment was dried until air-dry and then sieved through a sieve with a mesh diameter of 500 μm. Pots with a capacity of 1 L were prepared. In the pots, soil composite mixtures or biochar soil were placed on a layer of soil (400 g). The mixtures were prepared by mixing 400 g of soil with 15 g or 30 g of composites or biochar. The samples were marked as follows: B/30, B/15, BC1/30, BC1/15, BC2/30, BC2/15, BC5/30 and BC5/15. There were also 6 pots filled only with soil. The prepared substrates were left for 30 days. During this period, they were watered once every 7 days using 150 mL of distilled water.

After this time, spinach seeds were placed in the soil at a depth of 1.5 cm. Samples were prepared in three repetitions. The pots were placed in a phytotron chamber and left for 75 days, watered once every 5 days with 150 mL of water. Three pots filled with soil were watered alternately with water and a solution of NPK multi-component fertilizer (10 mL/1.5 L of distilled water). The operating parameters of the phytotron chamber were set as follows: temperature—24 °C, night duration—16 h, day duration—8 h and humidity—34%. After 75 days, the plants were cut down at soil level and dried in a laboratory dryer at 80 °C.

## 3. Results and Discussion

### 3.1. Selected Properties of Biochar and Fe-Biochar Composites

The yield of products produced in the pyrolysis process, i.e., biochar and BC1, BC2, BC3 and BC4 composites, is presented in Table 1.

The lowest production efficiency was recorded in the case of biochar production from sawdust. The pyrolysis temperature used leading to the carbonization of the product influenced, among other things, dehydrogenation, decarboxylation, deoxidation, dehydration and aromatization of the substrate [72]. The creation of composites was more efficient, varying depending on the type of additive and production method. Similar observations were reported by [73] who modified biochar with sodium pyrophosphate and also recorded a higher yield of such a composite compared to raw biochar. The highest efficiency was observed when creating the BC2 composite from biochar impregnated with FFHCO2. This was caused by a small loss of mass of biochar re-subjected to the pyrolysis process and a small loss of mass of the additive. A similar relationship occurred in the case of creating composites with the addition of iron salts. BC4 was produced with a higher yield than BC3. As reported by [74], the conversion of substrates into iron-containing biochar occurred with an efficiency of 80%. The reduction in efficiency may result from the loss of biochar during the process of chemical coprecipitation.

The produced biochar and composites were subjected to physico-chemical analysis. The results of this analysis are shown in Table 2.

The produced biochar had a pH of 5.1. The composites, depending on the production method and the additive used, had a pH in the range of 4.50–8.09. Composites made with the addition of iron salts had the lowest pH, which was probably caused by the pH of the prepared FeCl_2_ + FeCl_3_ solution.

The ash content in the analyzed samples varied and ranged from 2.00 to 78.36%. Biochar had the lowest ash content. Low ash content is characteristic of biochars of plant origin, unlike biochars from sewage sludge (79%) [75] or chicken manure (63.50%) [76]. The higher ash content in the composites was caused by the additives used in the production of the composites. The results obtained by [73] indicate a higher ash content in biochar composites with the addition of copper compounds. BC1 and BC2 composites prepared with the addition of FFHCO2 were characterized by the highest ash content (78.36% and 49.76%, respectively). Lower content was determined in BC3 and BC4 composites (24.72% and 12.33%, respectively) produced with the addition of iron salts. Lower ash content was recorded in BC2 and BC4 composites produced by double pyrolysis compared to BC1 and BC3 composites. Ash content may be an important feature of the sorbent because inorganic substances are involved in surface complexation [45].

Elemental analysis of the tested materials did not indicate the presence of nitrogen in any of them. This could be caused by the decomposition of nitrogen compounds at the pyrolysis temperature used. The carbon content ranged from 27.52 to 88.72%. Biochar had the highest carbon content. The content of this element was lower in the composites. The lowest values were determined for composites produced with the addition of FFHCO2. Perhaps the presence of this additive did not allow for greater carbonization of the produced composites. The C content in composites produced with the addition of iron salts ranged from 67.37 to 79.36%. Similar observations were reported by [59], who determined lower carbon content in biochar with the addition of FeCl_3_ produced at a temperature of 600 °C. Chen et al., 2011 [48] showed a decrease in the carbon content in magnetic biochar with increasing pyrolysis temperature.

The hydrogen content in the analyzed samples was in the range of 0.87–2.48%, and oxygen was 8.33–15.64%. This may indicate that the BC1 and BC2 composites have a larger number of functional groups containing oxygen compared to the others. Based on the percentages of C, H, O, H/C and O/C, ratios were determined, which, as indicated by [77], are correlated with the degree of aromaticity and polarity of biochar. The H/C value for all biochars was in the range of 0.28–0.38. The decreasing H/C value indicates increased aromaticity and carbonization of biochars [35,78]. In turn, increased aromaticity may indicate its stability and carbon sequestration capabilities [79]. According to [80] guidelines, the H/C value should not exceed 0.7. This criterion is met for biochar and all composites. The H/C ratio also indicates the potential of biochar to mitigate the effects of N_2_O emissions [81], with a product with an H/C value < 0.3 considered to be the most effective. The H/C value for the produced biochar and composites fluctuates around this value. Biochar produced at pyrolysis temperatures > 600 °C would be among the most effective in reducing N_2_O emissions [81]. According to [82], biochar with an O/C value < 0.2 is considered highly stable (half-life > 1000 years). This criterion is met by biochar and BC3 and BC4 composites. BC1 and BC2 composites are characterized by an O/C value ranging from 0.2 to 0.6, and therefore they can be described as moderately stable (half-life from 100 to 1000 years) [82]. The increasing O/C value indicates the increasing polarity and, at the same time, lower hydrophobicity of biochar [62]. Therefore, the BC1 composite seems to be the least hydrophobic of the tested samples. H/C and O/C atomic ratios for biochar produced by [77] at a temperature of 500 °C were in the ranges from 0.42 to 0.81 and 0 to 0.27, respectively, and were higher than in the case of our products (except BC1).

**Figure 2:**
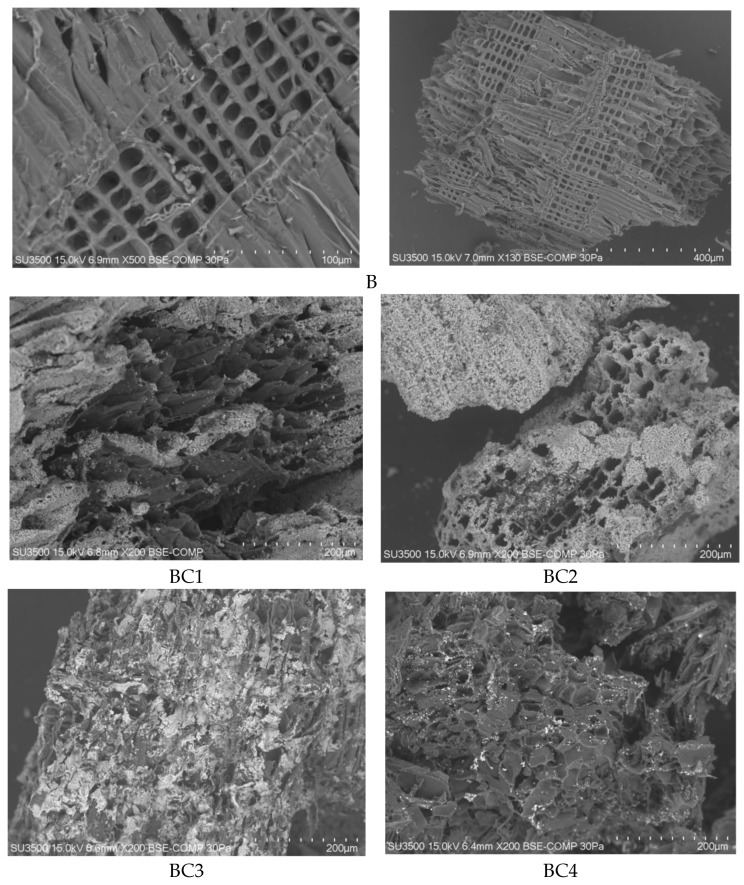
Microstructure of biochar (B) and composites (BC1, BC2, BC3, BC4).

Figure 2 shows the microstructure of the produced composites and biochar.

The photo presenting the microstructure of biochar (B) shows clear, regularly spaced pores and a “honeycomb” [83], a characteristic arrangement that may reflect the carbon skeleton of the capillary biological structure of the lignocellulosic raw material, because the biochar was produced from waste plant biomass. The BET area of biochar was 142.09 m^2^·g^−1^ (Table 2). In the study by [84], the specific surface area of biochar made of pine wood (pyrolysis temperature 525 °C) was determined to be 13.3 m^2^·g^−1^, which is definitely less than in our case. Similarly, Satyro et al. [61] determined the surface area of biochar from wood chips at the level of 4 m^2^·g^−1^, which increased in biochar modified with an iron solution to 25 m^2^·g^−1^.

The microstructure of the BC1 sample is clearly different, as it does not represent such a developed specific surface, which is reflected in the BET surface value of 95.14 m^2^·g^−1^. Sample BC2 has a similar microstructure, but is characterized by a higher specific surface area of 190.57 m^2^·g^−1^. Samples BC1 and BC2 are composites produced on the basis of biomass and waste FFHCO2. The additive used probably reduced the specific surface area of the BC1 composite, causing the pores to become clogged with FFHCO2 sediment. The procedure for producing the BC2 composite was different and this probably influenced the development of the specific surface at a level similar to that of biochar (B). The authors [85] indicate that filling the micropores with metal oxide deposits reduces the specific surface area of biochar. Both samples BC1 and BC2 show a characteristic white coating, which may represent some forms of iron compounds.

Sample BC3 had a specific BET surface of 179.23 m^2^·g^−1^. This composite was made on the basis of biomass impregnated with iron salts. The surface of the BC3 sample is heterogeneous and rough, with the presence of numerous cracks and cavities (Figure 2). This may indicate a large variation in pore sizes in the composites. The surface of the BC4 sample is similar. This may be the reason for the difference in the BET surface value characterizing samples BC3 and BC4. In the BC4 sample produced using a different method than the BC3 sample, the specific surface area could also have decreased due to the pores being blocked by iron compound particles. A similar situation was described by [47], the specific surface area was reduced in Fe-impregnated biochar as a result of blocking the pores with Fe_3_O_4_ formed in the pyrolysis process. The surface area of these biochars was smaller than in our studies, amounting to 2 m^2^·g^−1^, which was probably due to the substrate used for the production of biochar, which was MSW (municipal solid waste). Similarly, refs. [59,86] observed a reduction in specific surface area, pore diameter and total pore volume in iron-impregnated biochar when compared to raw biochar.

A white coating is visible on the surface of the BC3 composite, and on the surface of the BC4 composite, there are bright white spots that may be iron oxides. Mu et al., 2022 [87] observed a similar phenomenon in biochar enriched with iron.

**Figure 3:**
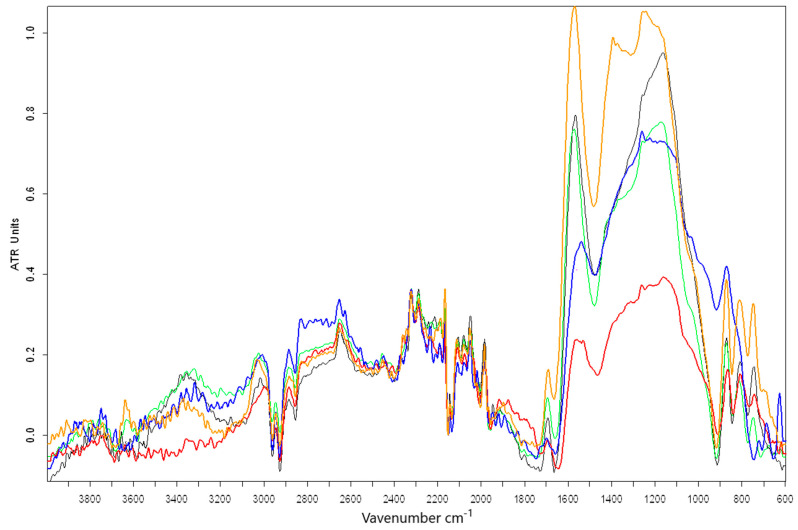
FTIR/ATR spectra of biochar B—yellow and composites: BC1—blue, BC2—red, BC3—green, BC4—black.

The analysis of FTIR spectra (Figure 3) obtained for biochar and BC1, BC2, BC3 and BC4 composites showed the presence of bands corresponding to the stretching vibrations of the C=O bond (in the region of a wavelength of 1691–1697 cm^−1^) in all analyzed samples, which indicates the potential presence of ketones, quinones and/or carboxylic carbon [28,74]. As [88] points out, the presence of C=O and other carbonyl or carboxyl groups can provide adsorption sites on the biochar surface.

Also, in all samples, the presence of stretching vibrations of the C=C bond was found in the wavelength range of 1531–1570 cm^−1^. Bonds of this type are usually broken down during the process carried out at temperatures of 600 °C and higher, which results in the loss of this type of structure in pyrolysis products [28,78]. The most intense peaks corresponding to these vibrations were marked for samples B, BC3 and BC4. Signals marked in this wavenumber range can also be associated with C=C vibrations in the aromatic ring, coupled to the carbonyl, ketone or ether group of C=O [12,89,90], which indicates the production of products containing in their structure organooxygen structural groups coupled with C=C double bonds in aromatic rings. The presence of functional groups containing oxygen may contribute to the formation of organometallic complexes and thus influence the sorption of metals [79,91,92].

However, the presence of deformation vibrations of the C-H bond was determined in samples B and BC2 in the area with a wavelength of 1393 cm^−1^. These may be areas corresponding to the CH methyl bond [62]. In all samples except BC4, the presence of stretching vibrations of the C-O bond was observed in the region of a wavelength of 1256–1260 cm^−1^. This may indicate the presence of aromatic ethers formed as a result of the inclusion of oxygen atoms in cyclic carbon structures [90]. The presence of C-O stretching vibrations in this wavelength range may also indicate the presence of lignin [93].

The presence of bands corresponding to the stretching vibrations of the C-O-C bonds was found in the BC2, BC3 and BC4 composites in the region of a wavelength of 1165–1171 cm^−1^. In all samples, the presence of bending vibrations of the =C-H bond was recorded, in areas with a wavelength of 866–871 cm^−1^. This may indicate the presence of polycyclic aromatic structures [28] in biochar and the produced composites.

In the analyzed spectra, other unidentified and not very intense peaks were observed in the area with a wavelength of approximately 3000 cm^−1^. The vibrations in this area may correspond to the stretching vibrations of the aliphatic CH_3_ and CH_2_ bonds [94]. Weak signals possibly corresponding to deformation vibrations or carbon dioxide in the C≡C plane were observed in the region with a wavelength of 2280 cm^−1^ [74].

In the analyzed spectra, no intense peaks were observed, probably corresponding to the stretching vibrations of the O-H bond or deformation vibrations of the O-H bond in the wavelength region of approximately 3100 cm^−1^. This was caused by the high temperature of biochar and composites production, which caused water loss and decomposition of volatile substances (instability of OH groups) in the analyzed samples. Carrying out pyrolysis at temperatures above 550 °C results in the reduction of oxygen functional groups [85]. As indicated by [85], metal oxides may affect the adsorption of pollutants, but they cause the degradation of functional groups containing oxygen located on the biochar surface.

### 3.2. Methylene Blue Sorption

Biochar and the produced composites were used in methylene blue sorption experiments. This dye is often used as an indicator of the sorption potential of biochars because of its cationic nature and large molecular size [95]. These features make it a good indicator of biochar’s ability to sorb heavy metal cations [96,97].

**Figure 4:**
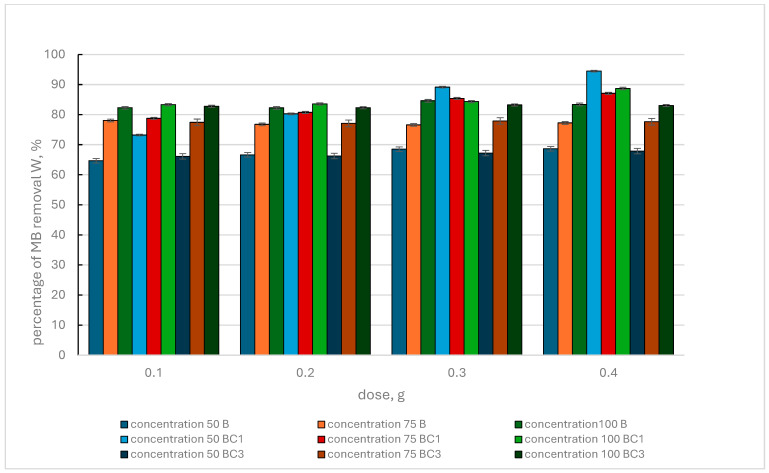
Percentage of methylene blue removal by biochar (B) and BC1, BC3 composites, depending on the initial MB concentration and adsorbent dose.

The results regarding the amount of dye removed from the solution by biochar and BC1 and BC3 composites (Figure 4) showed that at an MB concentration of 50 mg·L^−1^ and a dose of 0.1 g of adsorbent, the BC1 composite had the highest efficiency (73.22%).

The efficiency of dye removal for the BC3 composite was lower, but better than the efficiency of the use of biochar. At an initial concentration of 75 mg·L^−1^ MB and the same sorbent dose, the BC1 composite also turned out to be the most effective (78.77% MB removal). For the initial MB concentration of 100 mg·L^−1^, with the same adsorbent dose, the same relationship was observed, and the W value for BC1 was 83.31%. When using an increasing dose of adsorbent, the same trends in MB removal from the solution were observed. The maximum W value observed when using the BC1 composite was 94.99% (for a dose of 0.4 g). This was the maximum value obtained among adsorbents B, BC1 and BC3.

**Figure 5:**
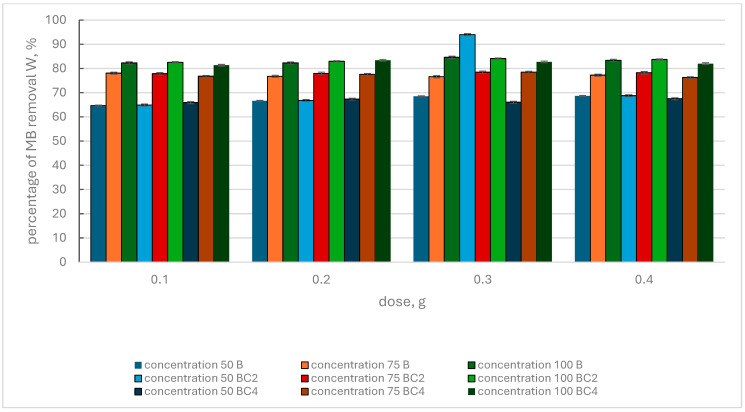
Percentage of methylene blue removal by biochar (B) and BC2, BC4 composites, depending on the initial MB concentration and adsorbent dose.

Comparing the results regarding the removal of MB from the solution using biochar and BC2 and BC4 composites (Figure 5), it can be seen that at an MB concentration of 50 mg·L^−1^ and an adsorbent dose of 0.1 g, the W value fluctuated in the range of 64.68–65.89%, without a clear dominance of one of the adsorbents.

At the MB concentration of 75 mg·L^−1^ and the same adsorbent dose, the maximum W value was recorded for B (78.07%), and the percentage of dye removal for BC2 and BC4 was 77.84 and 76.8, respectively. For a concentration of 100 mg·L^−1^MB, with the same adsorbent dose, the W value for B, BC2 and BC4 was at a similar level and amounted to 82.28%, 82.56 and 81.33%, respectively. Analyzing the results of MB removal from the solution for increasing doses of adsorbents and MB concentrations of 50 mg·L^−1^, 75 mg·L^−1^ and 100 mg·L^−1^, very similar trends were observed. The highest level of MB removal from the solution (84.61%) was observed for biochar, at a concentration of 100 mg·L^−1^ MB and a dose of 0.3 g. Taking into account the BC2 and BC4 composites, the highest W value was observed for the BC2 composite, which was 84.09% (concentration 100 mg·L^−1^ MB and adsorbent dose 0.3 g). The W value recorded for BC2, for a dose of 0.3 g of adsorbent and a concentration of 50 mg·L^−1^ MB, was probably a single departure from the observed trend.

Comparing the effectiveness of BC1 and BC3 composites produced according to one procedure but using different additives, the greater effectiveness of the BC1 composite is clearly visible. However, when comparing the effectiveness of removing MB from the solution by the BC2 and BC4 composites, a slightly better effect was noticed in the case of the BC2 composite. Analyzing the results obtained for BC1 and BC2 and BC3 and BC4 composites (Figure 4 and Figure 5), it was found that BC1 and BC2 composites have a greater potential for MB removal than BC3 and BC4 composites. Taking into account the results obtained using biochar, all adsorbents used can be ranked according to the maximum MB removal value achieved: BC1 (94.99%), B (84.61%), BC2 (84.09%), BC3 (83.23%) and BC4 (83.23%).

Researchers [98] developed a biochar composite containing Fe_3_O_4_ particles, which was characterized by a maximum MB adsorption capacity of 39.66 mg g^−1^. The sorption capacity of unmodified biochar was slightly lower and amounted to 23.17 mg g^−1^. In their research, Xie et al., 2021 [55] indicated that the introduction of iron oxide particles onto the biochar surface improved the adsorption capacity of MB. These authors used biochar from sorghum straw impregnated with iron chloride. Similar observations were presented by [99], who used a composite of biochar and iron oxide, and the tested MB removal capacity was increased. In our case, such a trend was observed only in the case of the BC1 composite. In turn, ref. [87] reports that sometimes the adsorption sites or pore structures of biochar may become occupied or blocked by iron oxide, which may lead to deterioration of adsorption capacity. Such a phenomenon could probably occur in the case of BC2, BC3 and BC4 composites. The authors [87] additionally applied KOH activation of the magnetic biochar used (specific surface area 409.15 m^2^·g^−1^), which allowed us to increase the adsorption efficiency (394.30 mg g^−1^). Adsorption without activation was small, indicating that the introduction of iron nanoparticles onto the biochar surface has little effect on the adsorption capacity. KOH promoted the formation of mesoporosity in the biochar. This is confirmed by research [100] that confirms the increased adsorption of MB on KOH-modified biochar. However, Samaraweera et al., 2023 [101] indicate that Douglas fir biochar modified with Fe_3_O_4_ showed a higher sorption capacity than raw biochar. These authors conclude that Fe_3_O_4_ nanoparticles facilitate the removal of large MB particles from the solution, and the large specific surface area (240 m^2^·g^−1^) favors physisorption. Similarly, authors [102] who used nano zero-valent iron-supported lemon-derived biochar indicate a highly effective MB adsorption process (1959.94 mg g^−1^).

### 3.3. Pharmaceutical Adsorption Experiments

#### 3.3.1. Removal of Amoxicillin from an Aqueous Solution

**Figure 6:**
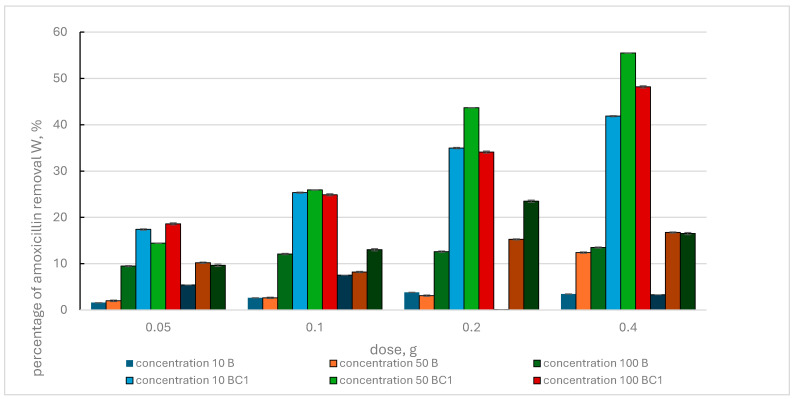
Percentage of amoxicillin removal by biochar (B) and BC1, BC3 composites, depending on the initial concentration of the pharmaceutical and the adsorbent dose.

Figure 6 shows the results of amoxicillin removal by biochar and BC1 and BC3 composites.

A general tendency was observed that the use of increasing doses of adsorbents and increasing concentrations of the pharmaceutical results in increased efficiency of amoxicillin removal from the solution. A departure from this tendency was found in the case of the BC3 composite, for which in some cases there was a slight decrease in the value of the W parameter.

The effectiveness of the BC1 composite was significantly higher than the effectiveness of the BC3 composite and biochar. The maximum values of amoxicillin removal by BC1 at increasing doses of this adsorbent and amoxicillin concentrations of 10 mg·L^−1^, 50 mg·L^−1^ and 100 mg·L^−1^ were 41.86%, 55.49% and 48.18%, respectively. However, for the BC3 composite, the corresponding values were 7.54%, 16.75% and 23.51%. The least effective was biochar, for which the maximum values of the W parameter at increasing doses of this adsorbent and amoxicillin concentrations of 10 mg·L^−1^, 50 mg L^−1^ and 100 mg·L^−1^ were equal to 3.82%, 12.42% and 13.50%, respectively.

**Figure 7:**
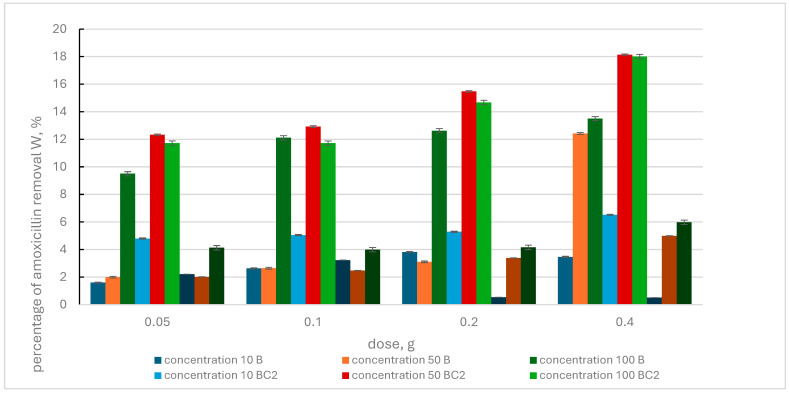
Percentage of amoxicillin removal by biochar (B) and BC2, BC4 composites, depending on the initial concentration of the pharmaceutical and the adsorbent dose.

Figure 7 presents the results of removing amoxicillin from an aqueous solution with biochar and BC2 and BC4 composites.

It was observed that with increasing sorbent dose and increasing pharmaceutical concentrations, the percentage removal of amoxicillin from the solution increased for individual adsorbents. The exception is the BC4 composite, for which a decrease in the W value was determined at the dose of 0.2 g and 0.4 g and the amoxicillin concentration of 10 mg L^−1^.

Comparing the effects of the composites, greater removal efficiency of this pharmaceutical was observed by the BC2 composite. At an amoxicillin concentration of 10 mg·L^−1^ and increasing doses of this composite, the W value was in the range of 4.8–6.52%. But already at the pharmaceutical concentration of 50 mg L^−1^ and 100 mg L^−1^ and increasing doses of the adsorbent, the W value was in the following ranges: 12.32–18.13% and 11.72–18.0%, respectively. However, the maximum percentage of pharmaceutical removal by the BC4 composite was 5.98% and was recorded at an amoxicillin concentration of 100 mg·L^−1^ and a composite dose of 0.4 g. The maximum efficiency of pharmaceutical removal for biochar was determined at the same pharmaceutical concentration and adsorbent dose, which was 13.50%.

Comparing the effectiveness of the BC1 and BC3 composites in removing amoxicillin from the solution, a greater sorption efficiency can be observed in the case of the BC1 composite. This effectiveness was also higher than when using biochar. When BC2 and BC4 composites were used, greater effectiveness of the BC2 composite was noted. The maximum percentage of amoxicillin removal from the solution achieved by the BC4 composite was lower than the maximum W value determined for biochar. Therefore, the tested adsorbents in terms of maximum efficiency of removing amoxicillin from the solution can be ranked as follows: BC1 (55.49%), BC3 (23.51%), BC2 (18.13%), B (13.50%) and BC4 (5.98%).

It can be assumed that composites produced using the double pyrolysis method, due to their properties (surface chemistry), have a lower antibiotic sorption capacity. Chen et al., 2019 [63] showed in their research that composites produced by pyrolysis of agricultural waste and then their impregnation have a 4-fold higher adsorption capacity than composites produced by impregnation and then pyrolysis.

Research results presented by [64] indicate that amoxicillin, both at low and high concentrations, can be effectively removed from the aqueous solution by the biochar composite. The adsorption capacity achieved by the authors was over 99 mg·g^−1^. However, in sorption tests ref. [64] used a composite produced at a temperature of 650 °C from banana biomass, impregnated with magnetic CoFe_2_O_4_ nanoparticles with a specific surface area of 190.5 m^2^·g^−1^.

A high antibiotic removal efficiency of 86% was achieved by [103]. However, the authors used modified Ag/Fe nanoparticles on a biochar carrier to remove cephalexin. Similar effectiveness of removing antibiotics such as azithromycin and ciprofloxacin (87.8%, 91.3%) using iron-modified biochar was achieved [74]. A high efficiency of 72.26% in removing amoxicillin from the solution was achieved by [104]. These researchers used ultrasound-functionalized corn cob biochar in their experiment. This adsorbent was characterized by a very large specific surface of 2368 m^2^·g^−1^. The adsorbent made of powdered pistachio shells covered with ZnO nanoparticles tested by [105] had a high sorption capacity of amoxicillin. Ajala et al., 2023 [14] extensively reported 98.1% removal of amoxicillin from aqueous solution on palm bark biomass with a 90 min contact time. Moreover, they indicated that activated carbons from olive seeds have a huge network of mesopores that enable them to sorb this antibiotic, and the sorption capacity of activated carbons modified with NH_4_Cl in relation to amoxicillin was 437 mg g^−1^—this effect was attributed to the large specific surface area, the presence of functional groups and interactions between the adsorbent and amoxicillin.

#### 3.3.2. Removal of Diclofenac from Aqueous Solution

**Figure 8:**
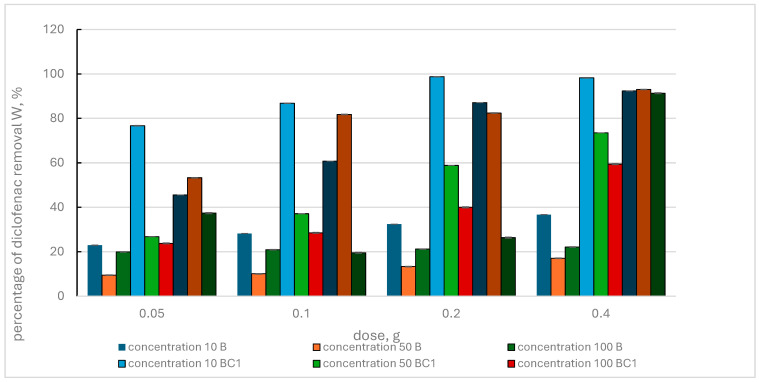
Percentage of diclofenac removal by biochar (B) and BC1, BC3 composites, depending on the initial concentration of the pharmaceutical and the adsorbent dose.

Figure 8 shows the results of the removal of diclofenac from aqueous solution by BC1, BC3 composites and biochar.

It was observed that with increasing sorbent dose and increasing pharmaceutical concentrations, the percentage of diclofenac removal from the solution increased for individual adsorbents. The exception is the BC3 composite, for which the reduced efficiency of removing the pharmaceutical from the solution was determined at the doses of 0.1 g and 0.2 g and the diclofenac concentration of 100 mg·L^−1^.

Comparison of the adsorption effect of BC1 and BC3 composites showed that the highest effectiveness was achieved with the BC1 composite, for which the maximum values of the W parameter, at a diclofenac concentration of 10 mg·L^−1^ and increasing doses of this pharmaceutical, were in the range of 76.70–98.71%. With a higher concentration of diclofenac in the solution and increasing doses of this adsorbent, lower removal efficiency of this pharmaceutical was observed. The maximum values of the W parameter recorded in this case were 73.47% and 59.42%. In the case of the BC3 composite, the highest value of the percentage removal of diclofenac that was determined at increasing doses of this adsorbent for individual pharmaceutical concentrations of 10 mg·L^−1^, 50 mg·L^−1^ and 100 mg·L^−1^ was, respectively, 87.08%, 82.39% and 37.39%.

When comparing the effectiveness of the adsorption effect of biochar to the effects of BC1 and BC3 composites, a clearly greater efficiency of diclofenac removal by the composites used (BC1 > BC3 > B) was noticed.

**Figure 9:**
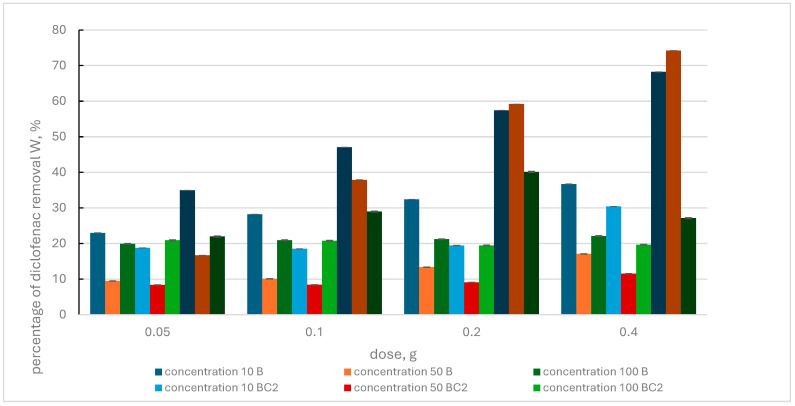
Percentage of diclofenac removal by biochar (B) and BC2, BC4 composites, depending on the initial concentration of the pharmaceutical and the adsorbent dose.

The analysis of the results of diclofenac removal by the BC2 composite (Figure 9) showed that increasing the adsorbent dose and increasing the concentration of the pharmaceutical did not cause any significant changes in the achieved effectiveness of BC2.

Only when a BC2 dose of 0.4 g and a diclofenac concentration of 10 mg·L^−1^ were used, the W parameter increased to a maximum value of 30.40%. An increase in the dose of the BC4 composite and the concentration of the pharmaceutical in the solution resulted in an increase in the value of the W parameter, except for one case in which the value of this parameter decreased (BC4 dose = 0.4 g, diclofenac concentration 100 mg·L^−1^). The effectiveness of the BC4 composite was higher than that of the BC2 composite. The maximum values of the W parameter achieved for increasing doses of the BC4 composite and pharmaceutical concentration: 10 mg·L^−1^, 50 mg·L^−1^ and 100 mg·L^−1^ were, respectively: 68.22%, 74.20% and 40.13%.

The highest efficiency of diclofenac removal by biochar was observed at a pharmaceutical concentration of 10 mg·L^−1^ and an increasing dose of this adsorbent. The W parameter values then ranged from 22.96% to 36.70%. The ranking of the adsorbents used in terms of effectiveness is as follows: BC4 > B > BC2.

The effectiveness of an increasing dose of adsorbent in removing diclofenac was demonstrated by [106]. These researchers used K_2_FeO_4_-activated sawdust biochar obtained in a one-stage carbonization and graphitization process [106]. Luo et al., 2019 [107] used microporous magnetic biochar (Fe/BC) with a large specific surface of 1986 m^2^·g^−1^. The adsorption capacity of diclofenac from an aqueous solution determined by the researchers was 361.25 mg g^−1^. Iron accumulated in biomass reached 4.6%.

Comparison of the results presented in Figure 8 and Figure 9 regarding the effectiveness of diclofenac removal by the manufactured composites and biochar allows us to rank the tested adsorbents in terms of maximum removal efficiency of this pharmaceutical from an aqueous solution: BC1 (98.71), BC3 (87.08%), BC4 (74.20%), B (36.70%), BC2 (30.40%)—(BC1 > BC3 > BC4 > B > BC2).

Lonappan et al., 2019 [84] described the results of research in which they showed greater efficiency of diclofenac removal by biochar from pig manure (88%) compared to biochar from pine wood (60–70%). However, the authors conducted column sorption experiments. The authors [12] investigated the use of modified biochar for diclofenac removal. The results of their research showed the high effectiveness of this adsorbent. However, the researchers used activation with NaOH. Adsorption experiments performed by [108] showed that a biomass-based biochar composite could remove 88.96% of diclofenac, (almost twice as much as raw biochar). However, the composite used is copper-enriched biochar (produced at 500 °C) having a higher specific surface area and total pore volume than raw biochar.

Anfar et al., 2020 [109] used iron oxide-enriched biochar prepared by microwave to evaluate the ultrasound-assisted adsorption capacity of salicylic acid, naproxen and ketoprofen. This adsorbent had a large specific surface of 786 m^2^·g^−1^, and the maximum adsorption capacities for the tested pharmaceuticals were high and amounted to 683 mg·g^−1^, 533 mg·g^−1^ and 444 mg·g^−1^, respectively.

### 3.4. Experiment for Removing Metals from Aqueous Solution

**Figure 10:**
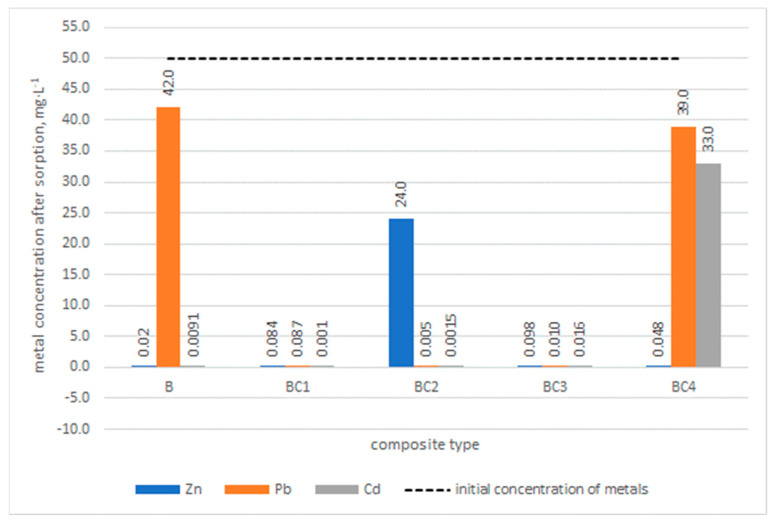
Final concentration of metals after the sorption process on biochar B and BC1, BC2, BC3, BC4 composites.

Analysis of the results of removing metals from aqueous solutions using biochar and BC1, BC2, BC3 and BC4 composites (Figure 10) showed that the best results in removing Zn, Cd and Pb were achieved when BC1 and BC3 were used.

The final concentration of the tested metals after sorption using these composites was less than 1% of the initial concentration. The use of the BC2 composite allowed for almost complete removal of Pb and Cd from the solution. The final concentration of Zn was determined at the level of 24 mg·L^−1^. The use of biochar to remove Zn and Cd resulted in the removal of these metals to the level of 0.02 mg·L^−1^ and 0.0091 mg·L^−1^, respectively. However, the Pb concentration remained at a high level of 42.00 mg·L^−1^. The BC4 composite turned out to be the least effective in removing metals. Its use turned out to be most effective for Zn removal. However, the final concentrations of Pb and Cd were high and amounted to 39 mg·L^−1^ and 33 mg·L^−1^, respectively.

The analysis of MB removal by the prepared composites and biochar showed that for the BC1 composite, over 90% removal of MB from the solution was found. In the case of the remaining composites, the maximum MB removal values were above 80%, but this is not reflected in the case of metal removal, except for the BC3 composite.

Taking into account the method of producing the composites, it can be observed that both the composite made of biomass impregnated with FFHCO2 and the composite made of biomass impregnated with iron salts had the best efficiency in removing metals from the solution. The common feature of these composites is the single-stage pyrolysis production method.

Kirmizakis et al., 2022 [62] indicate that iron modification played a significant role in the adsorption of As (removal rate 77.8%) by biochar. Satyro et al., 2021 [61] studied the adsorption of Se on biochar impregnated with ferric nitrate. The determined adsorption efficiency was 17.3 mg·g^−1^. Researchers [59] also described in their study the formation of new Se (VI) adsorption sites on Fe-modified biochar. Similar surface interactions during the adsorption of As (III) by Fe-impregnated biochar were indicated by [47]. As indicated by [47], better adsorption results were obtained by modifying biochar with Mg, and this adsorbent also had a higher specific surface area than the modified Fe.

Kashif Irshad et al., 2020 [110] showed that biochar modified with goethite limited the mobility of cadmium in the soil–plant system. Similar observations were reported by [111] who demonstrated the good ability of iron-enriched biochar to adsorb and immobilize the Cd ion. Li et al., 2021b [112] prepared a hybrid nanomaterial by impregnating biochar (containing high-density oxygen-charged groups) with nanoparticles of hydrated iron oxide. The resulting adsorbent is characterized by a higher Cd(II) sorption capacity compared to the basic material. Qian et al., 2022 [113] used nanoscale, porous biochar modified with zero-valent iron (BC-nZVI) to remediate soil enriched with lead and cadmium. The experimental results showed that the immobilization of Cd or Pb by BC nZVI was better (approx. 80%) than that of raw biochar. For Cr removal, ref. [114] used iron-containing biochar from ball milling. Iron oxides on the surface of biochars created Cr(VI) adsorption sites and therefore these composites showed increased Cr sorption potential. However, the sorption capacity of the biochar composite from pomelo peel and iron oxide towards Cr reached 24.37 mg g^−1^, as reported by [68,115] in their research, which showed greater efficiency of Cr (VI) removal by magnetic biochar with the addition of iron nanoparticles produced by wet and dry electromagnetic induction. Islam et al., 2021 [116] indicate that iron-enriched biochar reduced the average mobility of As (56%) and Cd (62%) in rhizosphere pore water, and supplementation with this composite reduces the accumulation of metals (loids) in rice tissues and increases grain yield.

### 3.5. The Impact of the Use of Biochar and Composites on the Growth and Yield of Plants

**Figure 11:**
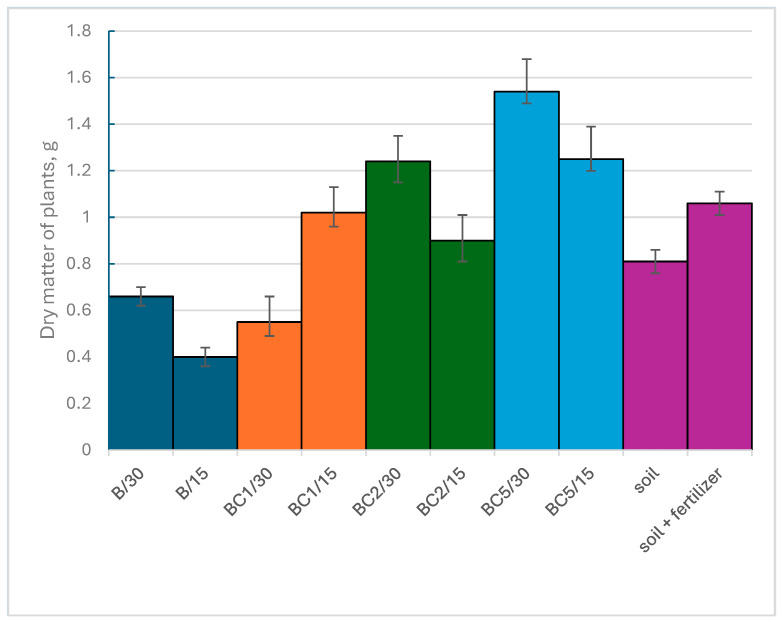
Dry matter of plants grown on prepared substrates.

Spinach is a leafy edible vegetable, so the plant’s biomass was assumed to be the entire plant cut from the pot above the soil layer. The mass of dried biomass of plants grown on prepared substrates is shown in Figure 11.

The analysis of the dry mass of plants showed that biomass growth is influenced by both the type of soil additive used and its dose. Comparing the growth of biomass on the substrate with the addition of biochar (B) and on the soil itself, it can be seen that the addition of biochar resulted in a slight reduction in the growth of plant biomass. Feeding the soil with fertilizer resulted in a greater increase in biomass than with the addition of biochar or the soil itself. When biochar-based composites were applied to the soil, the lowest biomass growth was determined for BC1 at a dose of 30 g. It was lower than the increase in biomass on soil alone and on the substrate with the addition of biochar. Reducing the dose of this composite had a significant impact on the growth of biomass. Better effects of biomass growth were observed when BC2 composite was added to the soil. The use of both doses in this case resulted in a greater increase in biomass than in the case of biochar or soil alone. However, the highest increase in biomass was recorded for the soil additive, which was the BC5 composite. In relation to the increase in biomass on soil, the use of BC5 at a dose of 30 g resulted in a 90% higher biomass increase, while at a dose of 15 g an increase in biomass growth was recorded by 54%. The effect of this composite was the best among the additives used for both doses. It was also greater than in the case of biomass growth on soil fed with fertilizer.

**Figure 12:**
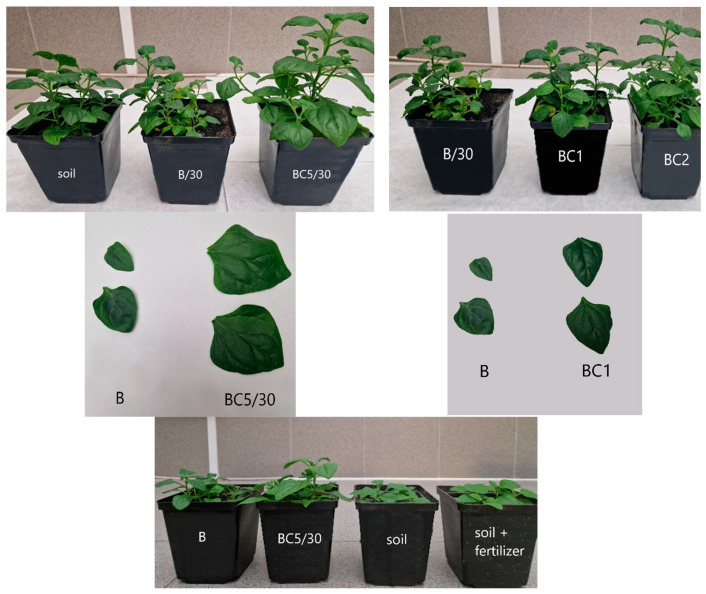
Growth and size of leaves of plants grown on various substrates.

Visual observations of the grown plants showed differences in their height, lushness and leaf size (Figure 12).

The smallest plants with small leaves grew on the B/30 substrate. The use of soil additives in the form of BC1 and BC2 composites resulted in the growth of slightly taller plants with slightly larger leaves compared to plants from B/30 substrates. The tallest, spreading plants with larger leaves were observed on BC5/30 and BC5/15 substrates. A greater increase in biomass occurred with an increased dose of this composite. Similar observations regarding plant growth and biomass growth are widely described in the literature, but most of them concern the use of unmodified biochars. Sikder and Joardar, 2018 [117] used biochar from poultry litter as a soil additive, characterized by an increased concentration of nutrients and noted increased plant growth and yield. Also, ref. [40] showed a positive effect of biochar on radish yield. Zhang et al., 2018 [118] indicated that adding biochar from manure to soil can increase yields and growth of upland barley in the short term.

In turn, ref. [111] used an iron-rich biochar composite, which improved radish growth and nutrient absorption. Chew et al., 2020 [119] used biochar enriched with nutrients, including Fe_2_O_3_ and FeSO_4_, which resulted in an increase in rice yield. Khan et al., 2022 [120] explained that iron-enriched biochar is an excellent soil additive for reducing the toxic effects of Ni, NaCl and CaCO_3_ stresses and improving wheat growth. Iron-enriched biochar used by [121] showed the greatest ability to improve soil quality and crop physiology among the tested additives.

In turn, the preparation developed by [122] had a positive effect on the growth of wheat at a low application rate. However, this composite contained a mixture of biochar, numerous minerals and a high concentration of magnetic iron (Fe) nanoparticles. Also, ref. [123] described the use of rice straw biochar with an admixture of iron oxide nanoparticles. The use of this composite significantly reduced the As content in the soil and improved the quality of soils and crops. Algethami et al., 2023 [124] successfully used iron-modified biochar for the re-cultivation of hazardous Cd and Pb-contaminated soils and for improving soil fertility.

## 4. Summary and Final Conclusions

The results of this research showed that the production of composites from waste plant biomass and FFHCO2 waste may be one of the directions for the use of these products. The use of FFHCO2 waste as an iron-introducing additive is definitely more economical than the use of commercial iron salts.

The results of experiments on removing contaminants from aqueous solutions indicate that the BC1 composite made of sawdust impregnated with FFHCO2 and subjected to the pyrolysis process has the greatest potential for sorption of MB, heavy metals (Zn, Cd and Pb), amoxicillin and diclofenac. However, confirmation of these relationships, stability and lack of toxicity of the BC1 composite requires detailed research.

The most positive effect on the growth and yield of biomass was determined in the case of the BC5 composite made of biochar impregnated with FFHCO2 and dried in an incubator. However, using the potential of this composite as a safe soil additive still requires further detailed research and verification of its long-term impact.

The production and use of BC1 and BC5 composites can be an economic strategy in waste management and CO_2_ sequestration processes.

## Figures and Tables

**Table 1:** Yield of biochar and Fe-biochar composites.

	B	BC1	BC2	BC3	BC4
Yield, %	25.2 ± 0.25	60.71 ± 1.90	95.97 ± 1.10	35.95 ± 1.40	65.18 ± 2.60

**Table 2:** Selected properties of biochar and composites.

Parameter		B	BC1	BC2	BC3	BC4	B5
pH		6.51	7.30	8.09	4.50	5.35	6.81
Ash	%	2.00	78.36	49.76	24.72	12.33	50.37
N	%	-	-	-	-	-	-
C	%	88.72	27.52	47.50	67.37	79.36	35.85 ^1^
H	%	2.48	0.87	1.24	2.11	1.91	nd ^2^
O	%	8.33	15.64	12.74	8.87	8.88	nd ^2^
H/C		0.33	0.38	0.31	0.38	0.28	nd ^2^
O/C		0.07	0.43	0.20	0.10	0.08	nd ^2^
BET	m^2^·g^−1^	142.09	95.14	190.57	179.23	93.06	nd ^2^

^1^ TC—total carbon, ^2^ nd—no data.

## Data Availability

Data are contained within the article.
